# Risk and protective factors for Long COVID in Brazilian adults (CUME Study)

**DOI:** 10.3389/fmed.2024.1344011

**Published:** 2024-02-21

**Authors:** Júlio Eduvirgem, Josefina Bressan, Helen Hermana Miranda Hermsdorff, Livia Cozer Montenegro, Marlise Lima Brandão, Alessandra Aparecida Tavares Neves, Lucas Samuel Aristides da Silva, Thiago Alexandre Gerake-Dias, Adriano Marçal Pimenta

**Affiliations:** ^1^Posgraduate Program in Nursing, Universidade Federal do Paraná, Curitiba, Paraná, Brazil; ^2^Department of Nutrition and Health, Universidade Federal de Viçosa, Viçosa, Minas Gerais, Brazil; ^3^Department of Nursing, Universidade Federal do Paraná, Curitiba, Paraná, Brazil

**Keywords:** COVID-19, Long COVID, risk factors, protective factors, cohort studies

## Abstract

**Background:**

Most people recover from COVID-19, however, between 5 to 20% have experienced new, recurring, or continuous health problems four or more weeks after being infected, a phenomenon called Long COVID, and whose reasons for its manifestation are incipient. Our objective was to analyse the risk and protective factors for Long COVID in Brazilian adults participating in the CUME Study.

**Methods:**

The CUME Study is a prospective cohort conducted with graduates from federal universities in the State of Minas Gerais, Brazil. In this study, 390 participants who answered the baseline questionnaire in 2016 and the third follow-up questionnaire in 2022 (which contained a block of questions about occurrence of COVID-19 and Long COVID) were included. The diagnosis of Long COVID was based on self-reporting of persistence of signs and symptoms of COVID-19 between 30 days and 6 months after remission of the disease. To estimate the risk and protective factors for Long COVID, a hierarchical multivariate statistical analysis was conducted using the Poisson regression technique.

**Results:**

Long COVID was observed in 48.9% of the participants. The following characteristics were identified as risk factors for the outcome: female sex (RR = 1.56; 95% CI = 1.22–1.99); prior diagnosis of hypertension (RR = 1.46; 95% CI = 1.19–1.80); having contracted COVID-19 in the first (RR =1.38; 95% CI = 1.07–1.79) or in the second waves (RR = 1.33; 95% CI = 1.07–1.65) of the pandemic period; and having presented three or more signs and symptoms during the acute phase of COVID-19 (RR = 2.99; 95% CI = 1.08–8.24). On the other hand, having a doctoral/postdoctoral educational level (RR = 0.69; 95% CI = 0.50–0.94) was identified as a protective factor for the outcome.

**Conclusion:**

Health system managers and healthcare professionals should be aware of the socioeconomic profile and disease history of patients who have had COVID-19 because women, people with a prior diagnosis of hypertension, and those who manifested multiple signs and symptoms of COVID-19 during the acute phase of the disease were at greater risk of developing Long COVID.

## Introduction

The coronavirus disease (COVID-19) is an infectious illness caused by the SARS-CoV-2 virus, whose first official case was recorded in the city of Wuhan (China) at the end of 2019 (1, 2). It spread throughout the world and was declared a pandemic by the World Health Organization (WHO) between March 11, 2020, and May 5, 2023 (3, 4).

COVID-19 manifests itself, in most cases, as a mild to moderate respiratory illness, and infected people recover without needing special treatment. However, some become seriously ill and may die (2). Official WHO data from October 25, 2023, indicated that there were 771,549,718 confirmed cases and that 6,974,473 deaths occurred from COVID-19 globally, with Brazil ranking sixth and third, respectively, in the number of confirmed cases (37,721,749) and deaths (704,659) (4).

Although we are in an endemic period and most people have recovered from the disease, between 5 and 20% have presented new, recurring, or continuous health problems four or more weeks after acute phase of COVID-19. This outcome has been called Long COVID and manifests as one or more of the following signs and symptoms: fatigue, headache, ringing in the ears, loss of smell, persistent cough, chest pain, inflammation of the heart, shortness of breath, palpitations, muscle aches, tingling sensation, diarrhoea, abdominal pain, rash, recurrent fever, forgetfulness, and depression (5).

The explanations for why some people develop Long COVID are still incipient, although they are associated with increased age, the number and severity of signs and symptoms during the acute phase of COVID-19, female sex, smoking, alcoholism, and prior diagnosis of chronic diseases (6–8). Furthermore, most scientific findings come from research conducted in high-income countries (6–8), and particularly in Brazil, they were conducted with samples of patients who were discharged after some period of hospitalization for COVID-19 (9, 10).

Therefore, conducting new studies on the subject becomes relevant because more subsidies must be generated so that health managers can improve and propose policies and programs aimed at combating COVID-19 and the resulting consequences from this disease, such as Long COVID.

Thus, the objective of this study was to analyse the risk and protective factors for Long COVID in Brazilian adults participating in the Cohort of Universities of Minas Gerais (CUME Study).

## Materials and methods

### CUME Study

The CUME is an open cohort epidemiological study conducted in Brazil since 2016 with alumni from seven universities in the state of Minas Gerais [UFMG (Federal University of Minas Gerais), UFV (Federal University of Viçosa), UFOP (Federal University of Ouro Preto), UFLA (Federal University of Lavras), UFJF (Federal University of Juiz de Fora), UNIFAL (Federal University of Alfenas), UFVJM (Federal University of Jequitinhonha and Mucuri Valleys)]. Its objective is to evaluate the impact of Brazilian dietary patterns and nutrition transition on chronic noncommunicable diseases.

The recruitment of participants is permanent, allowing a continuous sample size growth with each follow-up wave, which occurs every 2 years. Thus, previously recruited participants receive new questionnaires, while new participants receive the baseline questionnaire.

The project design, dissemination strategies and baseline first participants’ profile were detailed in a previous publication (11).

### Data collection

To the data collection, we fitted a virtual platform where participants have access to informed consent forms and questionnaires of the study. After accepting the content of informed consent form, the participants complete online questionnaire, according to their wave of data collection.

Although the CUME Study is a closed cohort, for this sub-study, we selected only UFMG and UFV alumni who graduated between 1994 and 2014, making this sub-study a closed cohort (Supplementary material). Between March and August 2016, the participants completed the baseline questionnaire which had two question blocks. The first block contained questions about socioeconomic aspects, lifestyle, morbidity, medication use, personal history of clinical and biochemical tests over the past 2 years and anthropometric data. The second block was a validated food frequency questionnaire (FFQ), containing a set of 144 food items separated into eight food groups [dairy, meat and fish, cereals and legumes, oils and fats, fruits, vegetables, beverages, other foods (food preparations, sugar, honey, sweets, etc.)] (12).

The first and second follow-up questionnaires were completed by the participants, respectively, between March and August 2018 (2-year follow-up) and March and August 2020 (4-year follow-up). These questionnaires contained the same first questions block of the baseline questionnaire. Moreover, the in the first follow-up questionnaire were included questions about eating habits, ability for self-care and access to health services, and in the second follow-up questionnaire were included questions about working conditions and standard disorders of sleep.

Finally, between March and October 2022, the participants completed the third follow-up questionnaire (6-year follow-up) which also contained the same first block of the baseline questionnaire. Additionally, due to the fact that the COVID-19 pandemic began in the interval between data collection of the second and third follow-up questionnaires, we decided to explore this topic by including questions about the occurrence of COVID-19, carrying out tests to detect COVID-19, symptoms of COVID-19, hospitalization due to COVID-19, occurrence of Long COVID, signs and symptoms of Long COVID, vaccination against COVID-19.

The follow-up questionnaires aim to assess changes in lifestyle, food consumption and general well-being of participants, in addition to allowing the diagnosis of new cases of chronic noncommunicable diseases and testing new contemporary hypotheses that are important in that context, such as the case of COVID-19 and Long COVID.

This study was conducted following the guidelines established in the Declaration of Helsinki and all procedures involving study participants were approved by the Research Ethics Committee of the UFMG (CAAE: 44483415.5.1001.5149). Informed consent was obtained from all participants.

### Participants

**Figure 1:**
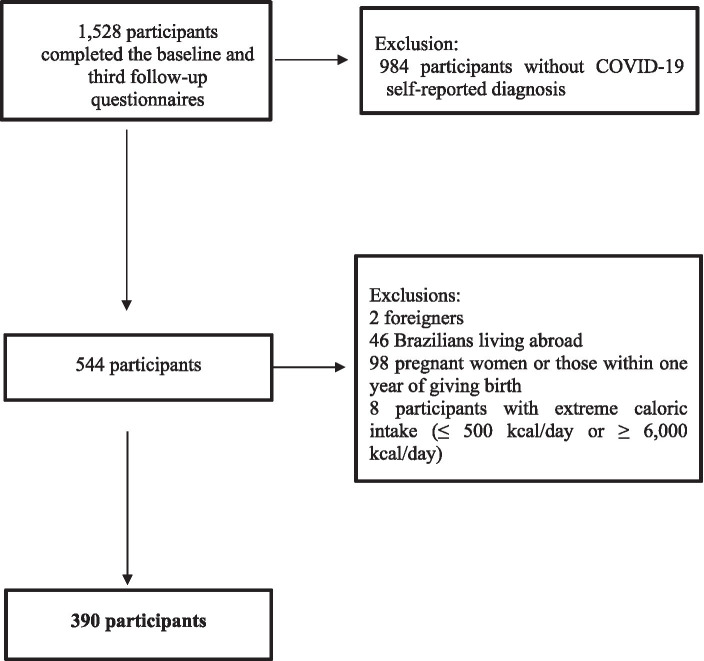
Flowchart of included participants. CUME Study, 2016–2022.

In this study, specifically, only the 1,528 alumni from UFMG and UFV who graduated between 1994 and 2014, and completed the baseline and all follow-up questionnaires were included (Supplementary material). Among them, we excluded 984 participants without COVID-19 self-reported diagnosis, two foreigners, 46 Brazilians living abroad, 98 pregnant women or those within 1 year of giving birth, eight participants with extreme caloric intake (≤ 500 kcal/day or ≥ 6,000 kcal/day) (13). Thus, the final sample included 390 participants (Figure 1).

### Outcome variable: diagnosis of Long COVID

In the third follow-up questionnaire (6-year follow-up), we included questions about Long COVID. One of these questions was: “In post-acute COVID-19 syndrome, clinical manifestations may last for several months after recovery from the infection. Check the main symptoms that you present or presented considering 30 days to 6 months after the end of the infection: intense fatigue; chronic pain; liver diseases; muscle weakness; difficulty breathing, cognitive deficits, such as changes in memory; neurological symptoms, such as loss of smell, dizziness and headaches; anxiety disorders and post-traumatic stress.”

If the participants did not indicate symptoms, they were considered without Long COVID (No); but if the participants checked one or more symptoms, they were considered to have Long COVID (Yes) (14, 15).

### Exposure variable: risk and protective factors for Long COVID

The exposure variables were: (1) the baseline characteristics of the participants regarding socioeconomic conditions [sex, age, skin colour, marital status, level of education, family income (minimum monthly salaries), area of professional training, and professional situation], lifestyle habits (smoking status, alcohol consumption, and physical activity), food consumption, self-reported health conditions [previous diagnosis of diseases (obesity, hypertension, type 2 diabetes mellitus, dyslipidaemias (hypercholesterolemia, hypertriglyceridemia, high blood levels of LDL-c, and low blood levels of HDL-c), asthma, and bronchitis)]; and (2) the 6-year follow-up characteristics of COVID-19 (signs and symptoms, severity, vaccination, waves).

The alcohol consumption was assessed according to binge drinking (drinking more than or equal to four doses of alcohol by women and more than or equal to five doses by men on a single occasion, considering the past 30 days) (16). Binge drinking was initially categorized into yes or no. Participants who answered “yes” were asked how many days of the month they were exposed to binge drinking (1 to 2 days/month, 3 to 4 days/month, and 5 or more days/month).

Physical activity was assessed by a list containing 24 leisure activities, described in minutes per week. Initially, it was categorized into light, moderate, and vigorous, and then the variable “level of physical activity” was created, categorized as “active” (≥ 150 min/week of moderate-intensity, ≥ 75 min/week of vigorous activity, or ≥ 150 min/week of vigorous and moderate intensity); “insufficiently active” (< 150 min/week of moderate-intensity, < 75 min/week of vigorous intensity, or < 150 min/week of vigorous and moderate intensity); and inactive (absence of physical activity during leisure time) (17).

Information on food consumption was investigated using the FFQ. Participants selected the food group items they consumed during the year before the survey and, when selecting food, they were asked to describe the size of the portions consumed in household measures (teaspoon, tablespoon, ladle, pinch, tong, saucer, cup, and glass) or traditional portions (units, slices, or pieces). Subsequently, the weekly, monthly, and annual intake frequencies of each food were transformed into daily consumption. Then, the daily food intake, in grams or millilitres, was calculated (serving size versus frequency of consumption).

The values of energy intake (kcal) and nutrients were calculated according to data provided in the Table of Measures Referred to Foods Consumed in Brazil (18), along with the Brazilian Table of Food Composition (19) and data from the United States Department of Agriculture (USDA) (20).

Then, the 144 food items in the FFQ were separated into groups according to the extent and purpose of industrial processing following the NOVA Classification (19): unprocessed/minimally processed foods (MPF), processed culinary ingredients (CI), processed foods (PF), and ultra-processed foods (UFP). In this study, unprocessed/minimally processed foods were grouped with processed culinary ingredients (MPF/CI) since the latter are not consumed on their own (21). Calorie contributions by the degree of processing were calculated from the sums of energy intakes of each food group, dividing the results by the total energy intake. These variables were divided into quintiles, with the first quintile used as the reference for data analysis.

Obesity was defined according to cut-off point proposed by WHO (Body Mass Index – BMI ≥ 30 kg/m2) (22). Hypertension was considered when the participants self-reported medical diagnosis of the disease or systolic blood pressure ≥ 140 mmHg or diastolic blood pressure ≥ 90 mmHg or use of antihypertensive (23). Type 2 diabetes mellitus also was considered when the participants self-reported medical diagnosis of the disease or glycemia ≥126 mg/dL or using oral antidiabetic or using insulin (24). Hypercholesterolemia, hypertriglyceridemia, high blood levels of LDL-c and low blood levels of HDL-c were identified when participants self-reported, respectively, cholesterol ≥190 mg/dL, triglycerides ≥150 mg/dL, LDL-c ≥ 130 mg/dL and HDL-c < 40 mg/dL (23). Finally, if the participants had hypercholesterolemia and/or hypertriglyceridemia and/or high blood levels of LDL-c and/or low blood levels of HDL-c, they were classified with dyslipidaemia (25).

In a previous study conducted with a sub-sample of the CUME Study, the self-reported data of weight, height, BMI, cholesterol, triglycerides, HDL-c, glycemia and blood pressure presented moderate to excellent agreement with those measured directly by the researchers. Moreover, the medical diagnosis of hypertension and the medical diagnosis of type 2 diabetes mellitus were also validated (26).

In Brazil, three waves of the pandemic period of COVID-19 have been described: the first from February 23, 2020 to November 7, 2020; the second from November 8, 2020 to December 25, 2021; and the third from December 26, 2021 to May 21, 2022 (27). Among our participants, four reported COVID-19 diagnosis in January 2020, and they were included in the first COVID-19 wave to data analysis. Moreover, 11 participants also reported COVID-19 diagnosis in June 2022, and they were included in the third COVID-19 wave to data analysis.

### Data analysis

Initially, the participants were characterized by presenting absolute and relative frequencies, means and standard deviations of their socioeconomic variables, lifestyle habits, food consumption, self-reported health conditions, and COVID-19, stratified by the occurrence or not of Long COVID. Statistical differences were evaluated using Pearson’s chi-squared test and *t*-Student test.

Next, to estimate the independent risk and protective factors for Long COVID, a hierarchical multivariate statistical analysis was conducted using the Poisson regression technique, dividing the variables into four blocks: (1) distal block = socioeconomic; (2) intermediate block 1 = lifestyle habits and food consumption; (3) intermediate block 2 = self-reported health conditions; and (4) proximal block = clinical characteristics of acute phase and vaccination against COVID-19. We chose to use Poisson regression technique because the participants have similar follow-up times, approximately 6 years (28, 29).

Thus, in the first stage, the variables that were associated with Long COVID at a statistical significance level of 20% in the bivariate analysis were selected for the final model. Then, each of the variables in the distal block was inserted into the final model in descending order of statistical significance and removed one by one using the backward method until only those with statistical significance levels below 5% remained. Next, the same process was done for the variables in the other blocks. Therefore, in the end, the variables from the previous block adjusted the variables from the subsequent block.

## Results

### Descriptive characteristics

There were higher frequencies of female participants (62.8%), aged between 30 and 39 (46.2%), without a stable relationship (52.1%), white (64.1%), with graduation/specialization level of education (50.3%), with professional training outside healthcare (65.9%), engaged in some professional activity (80.5%), and with a family income greater than 10 minimum wages (45.1%) were observed. Additionally, 8.5% were smokers, 45.1% reported binge drinking pattern of alcohol consumption, and 55.4% were physically active. The mean percentage energy intakes of MPF/CI, PF, and UFP were 65.2, 10.3 and 26%, respectively. Participants who reported Long COVID were more likely to be female, have a graduate/specialization level of education, and have higher and lower consumption, respectively, of MPF/CI and PF (Table 1).

**Table 1:** Socioeconomic and lifestyle characteristics of participants according to the diagnosis of Long COVID.

Characteristics	Long COVID		
	No (*n* = 199)	Yes (*n* = 191)	Total (*n* = 390)
*Socioeconomic*			
Female sex*	53.3	72.8	62.8
Age (years)			
20 to 29	25.1	24.6	24.9
30 to 39	45.2	47.1	46.2
40 to 49	20.1	22.5	21.3
50 to 68	9.6	5.8	7.7
White skin colour	64.8	63.4	64.1
Without stable union	49.3	55	52.1
Non healthcare professional	67.3	64.4	65.9
Level of education*			
Bachelor/Specialization’s degree	44.7	56	50.3
Master’s degree	30.7	29.3	30
Doctoral/Postdoctoral’s degree	24.6	14.7	19.7
Working	77.9	83.3	80.5
Family income (minimum monthly salaries)			
< 4	19.6	21.5	20.5
5 to 9	31.7	37.2	34.4
≥ 10	48.7	41.4	45.1
*Lifestyle*			
Smoking	9	7.9	8.5
Binge drinking (times/month)			
0	53.8	56	54.9
1 to 2	21.6	27.2	24.4
3 to 4	12.6	8.9	10.8
5 and more	12.1	7.9	10
Physical activity			
Sedentary	19.1	24.6	21.8
Insufficient	24.6	20.9	22.8
Active	56.3	54.5	55.4
*Food consumption (% of energetic contribution/day)*			
In natura and minimally processed foods/culinary ingredients*	63.6 (0.8)	66.8 (1.4)	65.2 (0.8)
Processed foods*	11.1 (0.4)	9.5 (0.4)	10.3 (0.3)
Ultraprocessed foods	26 (0.7)	25.9 (0.9)	26 (0.6)

CUME Study, 2016–2022. Data presented as percentage or medium (standard deviation). *p-value < 0.05 by Pearson’s chi-square or *t*-Student test.

The frequencies of the participants’ underlying pathologies were: 13.3% obesity; 11.5% hypertension; 2.1% type 2 diabetes mellitus; 46.7% dyslipidaemia (18% hypercholesterolemia, 10.5% hypertriglyceridemia, 11% high plasma concentrations of LDL-c, 26.4% low plasma concentrations of HDL-c). Additionally, 8.2 and 6.9% reported medical diagnoses of asthma and bronchitis, respectively. Participants who reported Long COVID were more likely to have hypertension (Table 2).

**Table 2:** Health and COVID-19 clinical conditions of participants according to the diagnosis of Long COVID.

Characteristics	Long COVID		
	No (*n* = 199)	Yes (*n* = 191)	Total (*n* = 390)
*Self-reported health conditions*			
Obesity	11.6	15.2	13.3
Hypertension*	7.5	15.7	11.5
Type 2 diabetes mellitus	2	2.1	2.1
Dyslipidemias	45.7	47.6	46.7
Hypercholesterolemia	16.1	19.9	18
Hypertriglyceridemia	9.1	12	10.5
High blood levels of LDL-c	12.1	10	11
Low blood levels of HDL-c	28.6	24.1	26.4
Asthma	8.5	7.9	8.2
Bronchitis	6	7.9	6.9
*COVID-19 clinical conditions*			
Number of infections			
1	88.9	86.4	87.7
2	10.6	10.5	10.5
3	0.5	3.1	1.8
Waves of the infection*			
First	13.1	17.3	15.1
Second	29.1	38.7	33.9
Third	57.8	43	51
Did some COVID-19 test	94.5	93.7	94.1
Symptoms*	92.4	98.4	95.4
Respiratory*	50.8	66	58.2
Fatigue*	46.7	68.6	57.4
Body temperature*	44.2	58.1	51
Headache*	37.2	64.9	50.8
Sore throat	45.7	49.2	47.4
Muscle pain*	36.7	58.6	47.4
Gastrointestinal	17.1	38.7	27.7
Number of symptoms*			
0	7.6	1.6	4.6
1 to 2	25.3	12.6	19
≥ 3	67.2	85.9	76.4
Searched for health care*	62	72.9	67.5
Hospitalization (*n* = 8)	1.5	2.6	2.1
1 to 7 days	100	80.8	85.7
8 to 23 days	0	9.2	14.3
Procedures during hospitalization	0	40	25
Oxygen by nasal catheter	0	40	25
Doses of vaccine			
0	1.5	1.6	1.5
1 to 2	9.6	12.6	11
≥ 3	88.9	85.9	87.4

CUME Study, 2016–2022. Data presented as percentage or medium (standard deviation).

*p-value < 0.05 by Pearson’s chi-square test.

Furthermore, it was found that 12.3% of the participants had contracted COVID-19 more than once, 51% was infected in the third wave, 94.1% were tested for the disease, and 95.4% presented symptoms, in the following order of magnitude: respiratory (runny nose, shortness of breath, wheezing, chest pain, others = 58.2%), fatigue (57.4%), body temperature (fever or chills = 51%), headache (50.8%), sore throat (47.4%), muscle pain (47.4%), gastrointestinal (nausea, abdominal pain, diarrhoea = 27.7%). Additionally, 67.5% of the participants sought health services and only 2.1% required hospitalization (85.7% within a week; 75% without the need for procedures; with the only necessary procedure being non-invasive mechanical ventilation). Regarding vaccination, 87.4% of the participants took three or more doses of COVID-19 vaccines. Participants who reported Long COVID were more likely to have manifested three or more symptoms of COVID-19, to have been infected in the second wave of pandemic period, and to have sought health services (Table 2).

### Frequencies of Long COVID and its signals and symptoms

Of the total 390 participants in the study, 191 reported signs and symptoms of Long COVID (48.9%), in the following order of magnitude: cognitive deficits, such as changes in memory (57.6%); intense fatigue (47.1%); neurological symptoms, such as loss of smell, dizziness, and headaches (36.7%); muscle weakness (35.1%); anxiety disorders and post-traumatic stress (22.5%); difficulty breathing (18.3%); chronic pain (13.1%); liver diseases (1.1%).

Among 42 participants who has been infected twice with COVID-19, 21 (50%) reported signs and symptoms of Long COVID, in the following order of magnitude: cognitive deficits, such as changes in memory (81%); intense fatigue (71.4%); neurological symptoms, such as loss of smell, dizziness, and headaches (57.1%); muscle weakness (38.1%); difficulty breathing (28.6%); anxiety disorders and post-traumatic stress (23.8%); chronic pain (23.8%).

Finally, six participants were infected with COVID-19 three times and all of them reported signs and symptoms of Long COVID, in the following order of magnitude: cognitive deficits, such as changes in memory (83.3%); intense fatigue (66.7%); neurological symptoms, such as loss of smell, dizziness, and headaches (50%); muscle weakness (50%); anxiety disorders and post-traumatic stress (50%); difficulty breathing (33.3%); chronic pain (33.3%).

### Independent risk and protective factors for Long COVID

Table 3 presents the results of the hierarchical multivariate model constructed using Poisson regression technique. Independent risk factors for Long COVID were female sex (RR: 1.56; 95% CI: 1.22–1.99), prior diagnosis of hypertension (RR: 1.46; 95% CI: 1.19–1.80), having contracted COVID-19 in the first (RR =1.38; 95% CI = 1.07–1.79) or in the second waves (RR = 1.33; 95% CI = 1.07–1.65) of the pandemic period, and having presented three or more symptoms of COVID-19 during the acute phase of the disease (RR: 2.99; 95% CI: 1.08–8.24). On the other hand, having higher education levels (doctorate/post-doctorate) constitutes an independent protective factor for Long COVID (RR: 0.69; 95% CI: 0.50–0.94).

**Table 3:** Hierarchical multivariate model of risk and protective factors for Long COVID.

Characteristics	Long COVID		
	RR	95% CI	*p*-value*
*Distal block*			
Sex			
Male	1 (Ref.)	–	–
Female	1.56	1.22–1.99	< 0.001
Level of education			
Bachelor/Specialization’s degree	1 (Ref.)	–	–
Master’s degree	0.89	0.71–1.11	0.311
Doctoral/Postdoctoral’s degree	0.69	0.50–0.94	0.020
*Intermediate block*			
Hypertension			
No	1 (Ref.)	–	–
Yes	1.46	1.19–1.80	< 0.001
*Proximal block*			
Waves of COVID-19 infection			
First	1.38	1.07–1.79	0.014
Second	1.33	1.07–1.65	0.009
Third	1 (Ref.)	–	–
Symptoms of COVID-19			
0	1 (Ref.)	–	–
1 to 2	1.84	0.64–5.32	0.260
≥ 3	2.99	1.08–8.24	0.034

CUME Study, 2016–2022. RR, relative risk; 95% CI, 95% confidence interval.

*p-value from Poisson regression; Bayesian Information Criterion (BIC) of Model 1 (variables of distal block) = 666.665; BIC of Model 2 (variables of distal block + variable of intermediate block) = 669.3825; BIC of model 3 (variables of distal block + variable of intermediate block + variables of proximal block) = 678.1191.

## Discussion

In this study, the frequency of Long COVID occurrence was high (48.9%), with its risk factors being female sex, prior diagnosis of hypertension, having contracted COVID-19 in the first or in the second waves of the pandemic period, and having presented three or more symptoms of COVID-19 during the acute phase of the disease. On the other hand, having a high education level (doctorate/post-doctorate) constituted a protective factor.

Longitudinal studies conducted with the general population in other countries have also shown a high frequency of Long COVID occurrence, ranging from 18.5% in the United States (30) to 84.7% in Israel (31). A meta-analysis on the subject estimated an average prevalence of the outcome at 64% (32). This wide variation in the proportion of people affected by Long COVID around the world may be influenced by structural issues of health services that affect access to treatment and prophylactic measures against COVID-19, as well as differences in the definition of Long COVID (7).

In this study, being female increased the risk of Long COVID by 56%. Our scientific findings are corroborated by results from several longitudinal studies conducted in Brazil and other countries that unanimously identified the female sex as a risk factor for Long COVID (33–42). The explanations for why women have a higher risk for developing Long COVID are still incipient. In general, middle-aged women are at a higher risk of presenting a series of debilitating continuous symptoms such as fatigue, shortness of breath, muscle pain, anxiety, depression, and “brain fog” after the acute phase of COVID-19 (43). Additionally, studies on COVID-19 have indicated that women exhibit more exacerbated humoral and cellular responses to the disease (43, 44) and this phenomenon could influence the persistence of signs and symptoms and trigger the occurrence of Long COVID (9).

Our results indicated that prior diagnosis of hypertension increased the occurrence of Long COVID by 46% which is like the findings of previous longitudinal studies conducted in France (45) and Saudi Arabia (46). A case–control study conducted with patients admitted to a hospital in Madrid, (Spain) due to COVID-19 during the first wave of the pandemic showed that pre-existing hypertension was associated with a greater number of Long COVID symptoms (47). During the acute phase of COVID-19, patients with cardiovascular diseases, including hypertension, had a higher risk of worsening clinical conditions and death from COVID-19 (48). This situation occurs potentially because there is an exacerbated pro-inflammatory response (e.g., cytokine storm) associated with the SARS-CoV-2 infection in people with hypertension mediated by the angiotensin-converting enzyme 2 (ACE2) receptor (47–49). Thus, this condition also influences the persistence of COVID-19 signs and symptoms after the disease remission period, which characterizes Long COVID (6, 8).

Also, presenting three or more symptoms during the acute phase of COVID-19 was a predictor of Long COVID in this study, increasing the outcome by a 2.9-fold risk. Previous longitudinal studies have also shown that the greater the number of COVID-19 symptoms during the acute phase of the disease, the higher the risk of developing Long COVID (33–35, 45, 50, 51). A study conducted in France with patients who were discharged after hospitalization for COVID-19 showed that the number of initial signs and symptoms was more important than the severity of the acute phase of the disease for the occurrence of Long COVID (45). In general, people who manifested COVID-19 signs and symptoms presented more severe clinical cases due to exacerbated humoral and cellular responses, and possibly, such immunological activity is a predictor of Long COVID (6, 8).

Regarding risk factors, having contracted COVID-19 in the first or in the second waves of the pandemic period increased the occurrence of Long COVID by 38 and 33%, respectively. These results were similar to those observed in a study conducted with Italian healthcare workers, which the risk for Long COVID was higher in participants infected in the first (OR = 2.16; CI 95% = 1.14–4.09) or in the second waves (OR = 2.05; CI 95% = 1.25–3.38) of the pandemic period after 30 to 60 days since the acute phase of COVID-19 (42).

These findings can be explained by the facts that in the first waves of COVID-19 in Brazil: (1) The predominant SARS-CoV-2 variants (wild, Alpha and Gamma) were more virulent, influencing both the acute phase of COVID-19 and the occurrence of Long COVID (6, 42). Death rates per 100 thousand inhabitants were 76.5, 214.7 and 46, respectively, in the first, second and third waves (27) and (2) COVID-19 vaccines were not yet available or vaccination coverage was still low. Vaccination began in January 2021 and vaccination coverage reached 70% in December 2021 (27), during the second wave. Therefore, the effects of vaccination were more evident in the third wave, reducing the severity of the acute phase of COVID-19 and, consequently, its sequelae, such as the Long COVID (27, 42).

Having higher education was the only protective factor against Long COVID identified in this study. Therefore, having a doctorate/post-doctorate education level decreased the risk of developing Long COVID by 31% compared to the risk of having a graduate/specialization education level. This scientific finding is very important because even in a sample of participants who are already considered to have high education levels compared to the general population, as they all have at least a degree in some professional area, being even more educated reduced the risk of Long COVID occurrence. Results from previous studies corroborate our scientific findings by demonstrating that, according to the reference category for data analysis, higher education was a protective factor against Long COVID (28), or low education was a risk factor for the outcome (52).

Explanations about the association between education and Long COVID vary from a more sociological to a more physiological perspective. In general, the level of education is a social determinant of health and a predictor of COVID-19 severity (53). Additionally, education influences a person’s ability to reflect on their own health and understand how to distinguish between signs and symptoms of pre-existing chronic diseases and COVID-19, and consequently, Long COVID (54). Thus, people with low education tend to over-report Long COVID signs and symptoms compared to people with higher education who are more parsimonious. Also, most of the time, more educated people engage in professional activities that stimulate the brain, which would result in a protective cognitive reserve against diseases that cause neurological damage (55) such as Long COVID (56), which is characterized by memory loss and is one of the most important signs and symptoms.

The term “cognitive reserve” is defined as the brain’s ability to optimize and maximize performance and functioning by recruiting specific networks and using alternative cognitive strategies to deal with brain damage or pathology (57). It is well documented in scientific literature that stimulating activities such as reading books, years of schooling, etc. would enhance neural resources, constituting the substrate of cognitive reserve that allows a person to attenuate cognitive decline resulting from aging or diseases that cause this outcome (57, 58).

Emphasizing that our participants with a doctoral/postdoctoral educational level were largely researchers and university professors and, therefore, engaged in more brain-stimulating activities that generate cognitive reserve than participants with less education.

### Study limitations and strengths

It is suggested that our scientific findings should be interpreted with caution due to some limitations: (1) The signs and symptoms of Long COVID were self-reported. However, studies conducted with a sample with high education, such as CUME, indicated excellent accuracy of self-reported data (59); (2) Our sample is small, not representative of the Brazilian population, and limited to participants with high education. However, our participants hold high and crucial positions for the Brazilian economy, and interruption of their work activities due to illness or death may result in significant social and economic burdens for the country; (3) All participants had mild cases of COVID-19, and most did not require hospitalization or invasive procedures during treatment; and (4) We believe that vaccination against COVID-19 has a protective effect on the disease and Long COVID, as demonstrated in previous study (42). However, in this study, participants did not inform the dates of their vaccine doses, making it impossible to verify whether vaccination occurred before or after acute COVID-19 infection or the manifestation of signs and symptoms of Long COVID.

As potentialities, it is highlighted that this study presents a longitudinal design, ensuring the causality of the associations found. Additionally, it was the first Brazilian study developed with a general target audience, not restricted to hospital discharges, broadening the understanding of the studied theme to a wider spectrum of the population.

## Conclusion

Finally, it is concluded that the occurrence of Long COVID is a high-magnitude event, constituting an important public health problem to be faced by health system managers and health professionals in the coming years after the end of the COVID-19 pandemic period.

Health system managers and health professionals should pay attention to the socioeconomic profile and disease history of patients who had COVID-19 because women, people with a previous diagnosis of hypertension, and those who manifested multiple signs and symptoms of COVID-19 in the acute phase of the disease had a higher risk of developing Long COVID. Additionally, health policies and programs that promote activities to increase cognitive reserve should be encouraged, as high education has been shown to be a protective factor against Long COVID.

## Data availability statement

The original contributions presented in the study are included in the article/Supplementary material, further inquiries can be directed to the corresponding author.

## Ethics statement

The studies involving humans were approved by Universidade Federal de Minas Gerais. The studies were conducted in accordance with the local legislation and institutional requirements. The participants provided their written informed consent to participate in this study.

## Author contributions

JE: Conceptualization, Investigation, Writing – original draft. JB: Conceptualization, Data curation, Funding acquisition, Investigation, Project administration, Writing – review & editing. HHMH: Conceptualization, Data curation, Funding acquisition, Investigation, Project administration, Supervision, Writing – review & editing. LCM: Writing – review & editing. MLB: Writing – review & editing. AATN: Writing – review & editing. LSAS: Writing – review & editing. TAGD: Writing – review & editing. AMP: Conceptualization, Data curation, Formal analysis, Funding acquisition, Investigation, Project administration, Supervision, Writing – original draft.
