# Supplementary material for: Risk and protective factors for Long COVID in Brazilian adults (CUME Study)
Source: Front Med (Lausanne). 2024 Feb 21;11:1344011. doi: 10.3389/fmed.2024.1344011 (PMC10919220; doi:10.3389/fmed.2024.1344011)
Supplement: Supplementary file 1 [file Image_1.pdf]

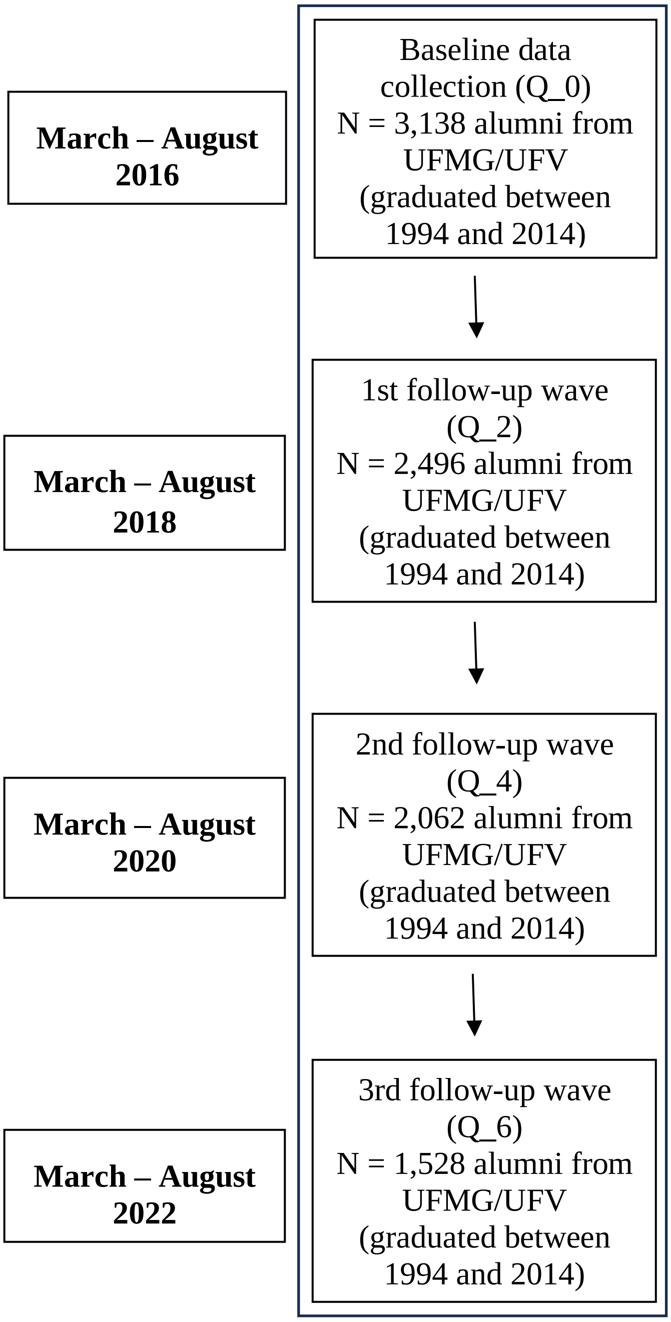

Supplementary material – Flowchart of data collection from the sample included in the present sub-study of the CUME Study, 2016-2022.

Note: Q_0 (baseline questionnaire); Q_2 (first follow-up wave = 2-year follow-up questionnaire); Q_4 (second follow-up wave = 4-year follow-up questionnaire); Q_6 (third follow-up wave = 6-year follow-up questionnaire); UFMG (Federal University of Minas Gerais); UFV (Federal University of Viçosa).
